# Development of osteoarthritis in adults with untreated hip dysplasia: a longitudinal observational study

**DOI:** 10.2340/17453674.2026.46828

**Published:** 2026-09-22

**Authors:** Rebecka VINGE, Cecilia ROGMARK, Daniel WENGER, Søren OVERGAARD, Carl Johan TIDERIUS

**Affiliations:** 1 Orthopaedics, Department of Clinical Sciences Lund, Lund University, Lund, Sweden; 2 Orthopaedics, Department of Clinical Sciences Malmö, Lund University, Malmö, Sweden; 3 Department of Orthopaedics, Skåne University Hospital, Lund and Malmö, Sweden; 4 Orthopaedic Surgery, Department of Clinical Medicine, University of Copenhagen, Copenhagen, Denmark;; 5 Department of Orthopaedic Surgery and Traumatology, Copenhagen University Hospital Bispebjerg, Copenhagen, Denmark

## Abstract

**Background and purpose:**

Although hip dysplasia is a risk factor for osteoarthritis (OA), its natural progression in adults remains poorly understood. In a longitudinal observational study, we aimed to estimate differences in OA incidence, time to OA, and minimum joint space width (JSW) between dysplastic hips and contralateral non-dysplastic hips.

**Methods:**

Adults aged 20–70 years who underwent pelvic radiographs in Malmö, Sweden, during 2007–2008 were included. Inclusion criteria were unilateral hip dysplasia (lateral center edge angle ≤ 20°), no OA at baseline, and available follow-up imaging of both hips. OA incidence was assessed using last available follow-up imaging, whereas minimum JSW was measured bilaterally on the last available radiograph of native hips.

**Results:**

50 participants (median age 47 years, IQR 39–62, 36 women) were included, with median follow-up 12.5 years (IQR 7.8–14.7). OA developed in 19/50 dysplastic hips and 16/50 non-dysplastic hips, corresponding to a paired risk difference of 6 percentage points (95% confidence interval [CI] –7 to 20). Mean time to OA was 8.2 years (SD 5.2) for dysplastic hips and 9.6 years (SD 5.1) for non-dysplastic hips (mean difference –1.2 years, CI –4.8 to 2.4). Minimum JSW at follow-up was 3.3 mm (SD 1.3) in dysplastic hips and 3.5 mm (SD 0.9) in non-dysplastic hips (mean difference –0.2 mm, CI – 0.6 to 0.2).

**Conclusion:**

No clear differences were observed in OA incidence, time to OA, or minimum JSW between dysplastic and contralateral non-dysplastic hips.

The prevalence of hip osteoarthritis (OA) is increasing steadily [1], and identifying modifiable risk factors is essential for the development of preventive treatments [2]. Adult hip dysplasia, characterized by insufficient acetabular coverage of the femoral head, is one such potentially modifiable risk factor [3]. Many cases are diagnosed in adulthood without a known history of developmental dysplasia of the hip (DDH) in childhood, suggesting that adult hip dysplasia may have an etiology distinct from DDH [4]. Previous studies have found that adult hip dysplasia is relatively common in the general population [5,6], and there is evidence supporting its role as a moderate risk factor for both radiographic [7] and clinically relevant hip OA [8], particularly in younger populations [9,10]. However, studies on the natural progression of OA in individuals with adult hip dysplasia remain scarce and have reported conflicting results [11-17]. Additionally, it is challenging to draw generalizable conclusions from the existing literature, as many studies do not clearly distinguish between individuals with a history of childhood DDH and those diagnosed in adulthood.

The aim of our study was to estimate differences in OA incidence, time to OA, and minimum joint space width (JSW) between dysplastic hips and contralateral non-dysplastic hips in adults with unilateral hip dysplasia.

## Methods

### Study design

This longitudinal observational study was based on imaging of the hips available in the radiographic records of the regional healthcare system in Skåne, Sweden. The study population consisted of adults with unilateral hip dysplasia, identified in a previous prevalence study [5]. Radiographic assessments were performed by a resident in orthopedic surgery, with support from 2 senior orthopedic consultants. The study was reported in accordance with the STROBE guidelines.

### Population

#### Dysplasia-cohort from prevalence study

In the previous prevalence study, 10,658 anteroposterior (AP) pelvic radiographs performed at Skåne University Hospital in Malmö between 2007 and 2008 were identified. Radiographs from individuals aged 20 to 70 years were then assessed for eligibility. The following exclusion criteria were applied: foramen obturator index outside 0.7–1.8 [18], OA (joint space narrowing), or other hip pathology, including a history of childhood hip disorder. After applying the exclusion criteria, 1,870 individuals remained for inclusion in the prevalence analysis. The lateral center–edge angle (LCEA) was measured on the AP pelvic radiographs as the angle between a line drawn from the center of the femoral head, perpendicular to the horizontal line, and another line drawn from the center of the femoral head to the lateral margin of the sclerotic zone of the acetabular roof (referred to as “the sourcil”). The horizontal line was drawn between the centers of the femoral heads [3]. Hip dysplasia was defined as an LCEA ≤ 20°. In the previous prevalence study, LCEA measurements were performed by a single observer, and intraobserver reliability of the LCEA measurements was assessed by repeating the measurements in 50 radiographs. The intraclass correlation coefficient (ICC) for the LCEA measurement was > 0.9, indicating excellent reliability according to Koo and Li [5]. A total of 98 cases of dysplasia were identified, of which 75 were unilateral and 23 were bilateral.

#### Inclusion in present study

Baseline imaging for the present study consisted of pelvic radiographs from the 98 participants previously identified with hip dysplasia [5]. The landmarks for the LCEA measurements were reassessed by the same observer who performed the original LCEA measurements, as a quality-control measure, and radiographic OA was reclassified using the Kellgren and Lawrence (K&L) classification [19] (< 2 or ≥ 2). Based on these revised baseline assessments, participants with unilateral hip dysplasia (LCEA ≤ 20° in the dysplastic hip and > 20° in the contralateral non-dysplastic hip) and no radiographic evidence of OA (K&L grade < 2) at baseline were included. The availability of follow-up imaging acquired between baseline and 2024 was then reviewed, and availability of follow-up imaging suitable for outcome assessment was required for inclusion in the study population. Imaging of any modality (plain radiographs, CT, and MRI) was included, provided that both hips were visualized and that the presence of radiographic OA, including total hip replacement (THR), could be determined. The patient administrative system was checked to determine whether participants had died or relocated outside the catchment area of the radiographic records. Participants who died during follow-up were eligible for inclusion, provided that suitable follow-up imaging before death was available. Baseline minimum JSW [20] was subsequently measured bilaterally on the pelvic radiographs of the included participants.

### Outcome

#### OA incidence and time to detection of OA

The last available imaging of the hips was used to assess presence of radiographic hip OA in both the dysplastic and non-dysplastic hips for each participant. THR was included as part of the OA outcome and was also reported separately. OA was assessed according to the K&L classification (< 2 or ≥ 2) on plain radiographs. For CT and MRI, the assessment followed the same rationale as the K&L scoring. In cases where OA was present, we reviewed earlier imaging to determine when OA could first be detected. In cases with THR, we confirmed that the surgery was performed due to OA, reported the date of surgery, and assessed when OA could first be detected before the procedure.

#### Minimum JSW

Minimum JSW at follow-up was measured on the last available plain radiograph or CT scan in which native hips were present bilaterally. This ensured that measurements for dysplastic and contralateral non-dysplastic hips were obtained from the same image, minimizing the impact of modality-related differences. Participants without suitable follow-up imaging of bilateral native hips were excluded from the minimum JSW analysis and remained included in analyses of OA incidence and time to OA.

### Statistics

Continuous variables are presented as means (SD) or medians (IQR), and categorical variables as counts and percentages. Baseline characteristics (age, sex, and reason for radiographic referral) were reported for participants with available follow-up imaging (included) and those without (excluded). Standardized differences were calculated to describe baseline imbalance between groups, using means (SD) for continuous variables and proportions for categorical variables.

Within-individual differences between dysplastic and contralateral non-dysplastic hips were estimated with 95% confidence intervals (CIs.) Continuous outcomes (baseline LCEA, baseline minimum JSW, time to OA, and minimum JSW at follow-up) were analyzed using paired t-test. Categorical outcomes (OA incidence and THR incidence) were analyzed as paired risk differences with 95% CIs (dysplastic hip minus contralateral non-dysplastic hip).

Statistical analyses were performed using IBM SPSS Statistics version 28 (IBM Corp, Armonk, NY, USA). Radiographs were stored, viewed, and measured using Sectra PACS (Sectra IDS7 version 26.1.5.7600; Sectra Medical, Linköping, Sweden). The LCEA measurements were performed using the dysplasia guide of the Sectra 2D Planning System (Sectra Orthostation Package version 10.1).

### Ethics, registration, data sharing plan, funding, and disclosures

The study was approved by the Swedish Ethical Review Authority (2023-04683-01). The previous prevalence study was performed with opt-out consent, and for this sequential study approval was granted to extend the review of the radiographic records without requiring further consent from the participants. The study was not registered in any database. As the data contains sensitive information, it is not publicly available in accordance with Swedish legislation. Funding was received from the Greta and Johan Kock Foundation. CR has received lecture fees from LINK and Swemac. Otherwise, the authors declare no conflicts of interest. Complete disclosure of interest forms according to ICMJE are available on the article page, doi: 10.2340/17453674.2026.46828

## Results

### Population

**Figure 1:**
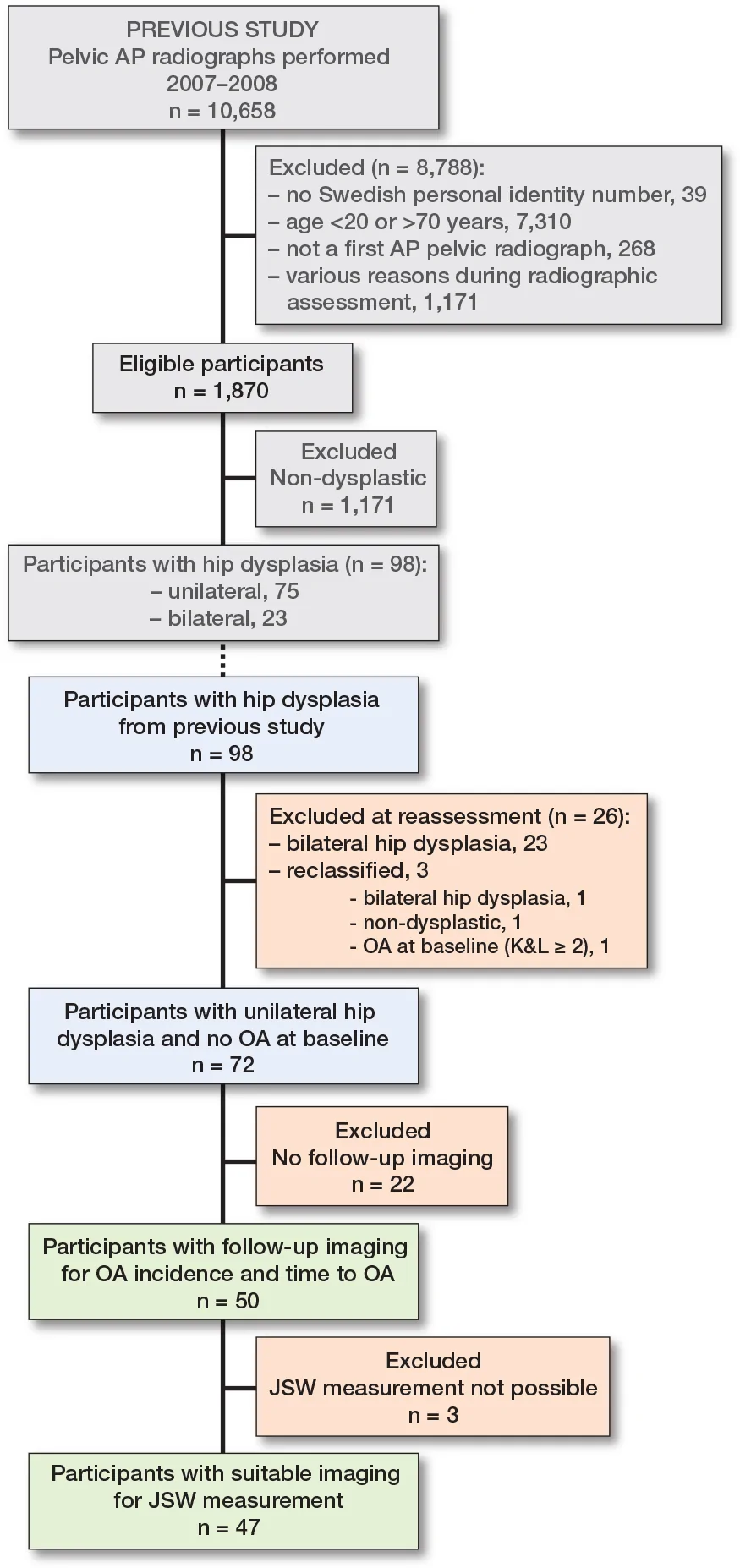
Flowchart of participant inclusion. Each included participant with unilateral hip dysplasia contributed 1 dysplastic hip and 1 contralateral non-dysplastic hip, enabling paired within-individual analyses. Analyses of minimum joint space width (JSW) were restricted to participants with suitable imaging. AP: anteroposterior, OA: osteoarthritis.

Of the 75 individuals with unilateral hip dysplasia identified in the prevalence study, 3 were excluded after reassessment: 1 had LCEA ≤ 20° bilaterally, 1 had an LCEA > 20° bilaterally, and 1 had K&L grade 2 at baseline. This left 72 unilateral cases eligible for follow-up. Among these, 50 individuals had available follow-up imaging suitable for assessing OA incidence and were considered the main study population. Of these 50, 47 had follow-up imaging where JSW could be measured bilaterally and were included in the JSW analysis (Figure 1). 12 participants died during the study period, with a mean time to death of 9.3 years (SD 4.4). No participants relocated outside the catchment area of the radiographic records. The median age at baseline was 47 years (IQR 39–62). Baseline data for both included and excluded (unilateral hip dysplasia without available follow-up imaging) participants, alongside standardized differences, are presented in Table 1. For included participants, baseline measurements of LCEA and minimum JSW are presented separately for the dysplastic and contralateral non-dysplastic hips (Table 2).

**Table 1:** Baseline characteristics of participants with unilateral hip dysplasia, according to follow-up availability, with standardized differences between groups

Baseline values	Participants with unilateral hip dysplasia (n = 72)		Standardized difference
	Included ^a^ (n = 50)	Excluded ^b^ (n = 22)	
Age, median (IQR)	47 (39–62)	45 (34–55)	0.21
Female sex, n	36	11	0.45
Trauma referral, n	18	10	0.18

a available follow-up;

b unavailable follow-up.

IQR = interquartile range.

**Table 2:** Lateral center–edge angle (LCEA) and minimum joint space width (JSW) at baseline for all included participants with unilateral hip dysplasia and available follow-up imaging, presented separately for dysplastic and contralateral non-dysplastic hips

Baseline values	Participants with unilateral hip dysplasia with available follow-up (n = 50)		
	Dysplastic side (n = 50)	Non-dysplastic side (n = 50)	Mean difference (CI)
LCEA °, median (IQR)	18.5 (17–20)	25 (23–28)	–7.5 (–8.5 to –6.4)
Minimum JSW, mm, mean (SD)	4.3 (0.9)	4.0 (0.7)	0.2 (0.1 to 0.4)

CI: 95% confidence interval; IQR: interquartile range.

### Outcome

#### OA incidence and time to detection of OA

**Figure 2:**
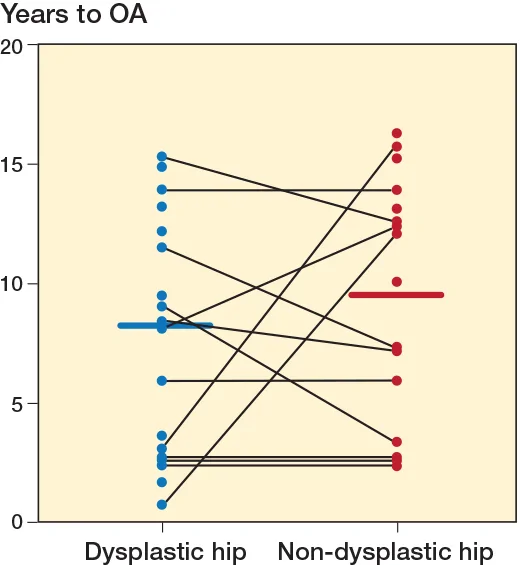
Univariate scatterplot for all values of time to detection of osteoarthritis (OA) for dysplastic (blue, n = 19) and non-dysplastic hips (red, n = 16). In cases where OA developed bilaterally (n = 12), the values of each subject’s dysplastic and non-dysplastic hip are connected by a black line. Mean time to OA for dysplastic hips was 8.2 years (SD 5.2) (blue horizontal line). Mean time to OA for non-dysplastic hips was 9.6 years (SD 5.1) (red horizontal line).

The most common imaging modality used for OA assessment was anteroposterior pelvic radiography (n = 21), followed by CT (n = 15) and spine MRI (n = 6). Other modalities were CT urography (n = 3), urography (n = 2), abdominal radiography (n = 2), and CT angiography of the aorta (n = 1). The median time from baseline to OA assessment was 12.5 years (IQR 7.8–14.7). OA was detected in 19/50 dysplastic hips and 16/50 non-dysplastic hips, corresponding to a paired risk difference of 6 percentage points (CI –7 to 20). In a descriptive subgroup analysis of the non-dysplastic hips, OA was detected in 8/28 hips with an LCEA of 21–25° and in 8/22 hips with an LCEA > 25°. The mean time to OA was 8.2 years (SD 5.2) for dysplastic hips and 9.6 years (SD 5.1) for non-dysplastic hips (Figure 2). Among the 12 individuals with bilateral OA, the mean within-individual difference was –1.2 years (CI –4.8 to 2.4). THR was performed in 5/50 dysplastic hips and 4/50 non-dysplastic hips. The median time to THR was 5.8 years (IQR 4.4–8.6) for dysplastic hips and 10.7 years (IQR 4.5–15.2) for non-dysplastic hips. Differences in THR incidence and time to THR were not estimated due to the small number of bilateral cases.

#### Minimum JSW

**Figure 3:**
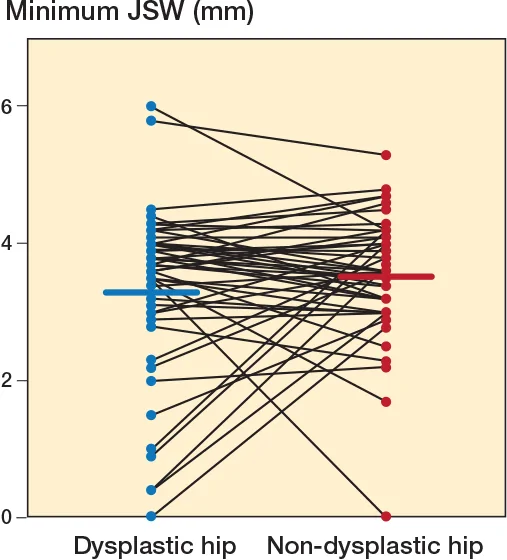
Univariate scatterplot of all measurements of minimum joint space width (JSW) for dysplastic (blue, n = 47) and non-dysplastic hips (red, n = 47), performed on the last available radiograph in which the participant had native hips bilaterally. Measurements for each subject’s dysplastic and non-dysplastic hip are connected by a black line. Mean minimum JSW for dysplastic hips was 3.3 mm (SD 1.3) (blue horizontal line). Mean minimum JSW for non-dysplastic hips was 3.5 mm (SD 0.9) (red horizontal line).

The median time from baseline to the last available imaging in which the minimum JSW could be measured bilaterally was 11.6 years (IQR 6.2–14.0). For 9 participants, the image used for JSW measurement was taken at an earlier timepoint than the image used for OA assessment. The mean minimum JSW was 3.3 mm (SD 1.3) for dysplastic hips and 3.5 mm (SD 0.9) for non-dysplastic hips, corresponding to a mean difference of –0.2 mm (CI –0.6 to 0.2) (Figure 3).

## Discussion

We aimed to estimate differences in OA incidence, time to OA, and minimum joint space width (JSW) between dysplastic hips and contralateral non-dysplastic hips in adults with unilateral hip dysplasia.

We found that differences in OA incidence, time to OA detection, and minimum JSW between dysplastic and contralateral non-dysplastic hips were small, and the 95% CIs were not suggestive of clinically relevant differences. To our knowledge, this study is the first to examine OA development in adults with hip dysplasia using contralateral non-dysplastic hips as controls.

The LCEA was measured to the lateral margin of the sourcil, which may yield slightly different values compared with measurements to the bony acetabular edge. This should be considered when comparing results with previous studies, as different definitions of the lateral margin may contribute to variability in reported thresholds and outcomes [21]. In addition, different LCEA threshold values have been used in the literature, which may further affect comparability across studies.

Our findings are consistent with those of a prospective case-control study, which found no clear difference in reduction of minimum JSW between dysplastic hips (LCEA ≤ 20°) and control hips (LCEA ≥ 25°) [14]. In contrast, a retrospective study with a longer follow-up of 20 years found that dysplastic hips (LCEA < 25°) had a higher risk of OA progression than hips with normal morphology (≥ 25°) [16]. However, the population in that study may have been at higher risk of OA development and progression, as all participants had already undergone THR in the contralateral hip at the time of inclusion. Other longitudinal studies exploring OA development in adult cohorts with hip dysplasia are limited by important methodological flaws. Most notably, they lack control groups, and several include individuals with a history of childhood DDH or pre-existing OA at baseline [11-13,15,17].

In our study, approximately 1/3 of both dysplastic and non-dysplastic hips developed incident radiographic hip OA. It is difficult to place these incidence estimates into a broader context, as study populations and OA definitions vary across previous studies on OA incidence [22]. For instance, in a cohort representative of the general population aged 55 years and older, with a mean baseline age of 66 years (SD 6.5), 1/5 of the participants developed radiographic OA after 7 years of follow-up [23]. In contrast, in a cohort of participants with recent onset of hip pain and a mean baseline age of 55.5 years (SD 5.4), half of the hips developed radiographic hip OA after 10 years [8].

Taken together, the prognosis of adult hip dysplasia may not be as poor as previously hypothesized. However, there is a need for prospective observational studies intended to longitudinally track the natural history of hip dysplasia in young adults without other risk factors for OA. Preferably, these studies should include both symptomatic and asymptomatic participants, as well as control groups, and incorporate clinical data such as symptoms, function, and quality of life.

### Limitations

Some baseline differences were observed between participants with and without available follow-up imaging, particularly in sex distribution. However, as the analyses were based on within-individual comparisons between hips, the impact of these baseline differences on the study results is likely to be small.

The assessment of hip OA in our study was limited to imaging available in the medical records. We cannot exclude the possibility that some participants may have developed radiographic OA without undergoing imaging during the follow-up period. In addition, follow-up duration varied between participants, and no data on hip-related symptoms was collected, limiting interpretation of the clinical implications of these findings. Some participants died during follow-up, and although their last available imaging was used for outcome assessment, death may have precluded development of OA and influenced the observed outcomes.

Imaging-based assessments relied on 2-dimensional representations and therefore provide a limited depiction of the hip joint’s 3-dimensional mechanical properties relevant to both acetabular morphology and hip OA. Furthermore, follow-up imaging included several modalities obtained for different clinical indications, where the hips were not always the primary focus of the examination. As these modalities differ in their technical characteristics, this may have influenced the assessment of OA and absolute measurements such as minimum JSW. However, as differences between dysplastic and contralateral non-dysplastic hips were estimated within individuals and based on the same imaging examination, the impact of imaging modality on the estimated differences is likely minimal. Minimum JSW at follow-up could not be assessed in 3 participants due to lack of suitable follow-up imaging with bilateral native hips, and analyses were therefore based on a subset of the cohort. This may have reduced precision but is unlikely to have introduced systematic bias. The relatively small sample size may have limited the precision of the estimates and larger studies are needed to confirm these findings.

Notably, the non-dysplastic hips in our cohort had a median LCEA of 25°, which is lower than normative values reported in previous population-based studies [5,24] and falls within the range that some authors consider borderline hip dysplasia [25]. Therefore, our results may not be generalizable to populations with greater acetabular coverage in their non-dysplastic hips. However, in a descriptive subgroup analysis of non-dysplastic hips, OA incidence appeared similar in hips with an LCEA between 21º and 25° and those with an LCEA > 25°. Finally, we cannot exclude the possibility that OA incidence in the non-dysplastic control hips was influenced by an underlying predisposition to bilateral OA in individuals with unilateral hip dysplasia.

### Conclusion

In a cohort of Swedish adults with unilateral hip dysplasia, no clear differences were observed in OA incidence, time to OA, or minimum JSW between dysplastic and contralateral non-dysplastic hips.

## Notes

RV: study design, data acquisition, statistical analysis, interpretation of data, manuscript writing. CR, SO: study design, interpretation of data, critical review of manuscript. DW: interpretation of data, critical review of manuscript. CJT: study design, supporting role during data acquisition, interpretation of data, critical review of manuscript. All authors: final approval of the version to be published and agreement to be accountable for all aspects of the work.

The authors thank Aleksandra Turkiewicz for statistical support.

Handling co-editors: Ilkka Helenius and Robin Christensen

*Acta* thanks Terje Terjesen and other anonymous reviewers for help with peer review of this manuscript.
